# Targeted β^−^-Particle Plus Conversion and Auger-Electron Therapy with ^161^Tb-Labeled Somatostatin Receptor Antagonist DOTA-LM3: A Phase 0 Study

**DOI:** 10.2967/jnumed.125.270654

**Published:** 2026-03

**Authors:** Julia G. Fricke, Frida Westerbergh, Lisa McDougall, Chiara Favaretto, Emanuel Christ, Guillaume P. Nicolas, Susanne Geistlich, David E. Schmid, Francesca Borgna, Melpomeni Fani, Peter Bernhardt, Nicholas P. van der Meulen, Cristina Müller, Roger Schibli, Damian Wild

**Affiliations:** 1 Division of Nuclear Medicine, University Hospital Basel, Basel, Switzerland;; 2 Department of Medical Radiation Sciences, Institute of Clinical Sciences, Sahlgrenska Academy at University of Gothenburg, Gothenburg, Sweden;; 3 Center for Radiopharmaceutical Sciences, PSI Center for Life Sciences, Villigen-PSI, Switzerland;; 4 ENETS Center of Excellence for Neuroendocrine and Endocrine Tumors, University Hospital Basel, Basel, Switzerland;; 5 Division of Endocrinology, University Hospital Basel, Basel, Switzerland;; 6 Division of Radiopharmaceutical Chemistry, University Hospital Basel, Basel, Switzerland;; 7 Department of Medical Physics and Biomedical Engineering, Sahlgrenska University Hospital, Gothenburg, Sweden;; 8 Laboratory of Radiochemistry, PSI Center for Nuclear Engineering and Sciences, Villigen-PSI, Switzerland; and; 9 Department of Chemistry and Applied Biosciences, ETH Zurich, Zurich, Switzerland

**Keywords:** peptide receptor radionuclide therapy, ^161^Tb, DOTA-LM3, DOTATOC, gastroenteropancreatic neuroendocrine tumors

## Abstract

The goal of this phase 0 study was to determine the absorbed doses in tumors and relevant organs after a test injection of [^161^Tb]Tb-DOTA-LM3 and [^177^Lu]Lu-DOTATOC in the same cohort of patients with grade 1 and 2 somatostatin receptor–positive gastroenteropancreatic neuroendocrine tumors. **Methods:** In this randomized, crossover, prospective, single-center, open-label phase 0 study, 8 patients received 1 GBq of [^161^Tb]Tb-DOTA-LM3 and 1 GBq of [^177^Lu]Lu-DOTATOC, with a 4-wk interval between injections. Quantitative SPECT/CT imaging was performed 3, 24, 72, and 168 h after administration of each radiopharmaceutical to calculate tumor and organ absorbed doses (3-dimensional dosimetry using a Monte Carlo–based ordered-subset expectation maximization algorithm). **Results:** After injection of 1 GBq of [^161^Tb]Tb-DOTA-LM3, SPECT/CT revealed excellent image quality with intense tumor uptake in all patients and a median of the mean effective tumor half-life of 103 h (range, 56–152 h) for [^161^Tb]Tb-DOTA-LM3 and 83 h (range, 30–122 h) for [^177^Lu]Lu-DOTATOC (*P* = 0.012). The medians of the mean tumor absorbed doses of [^161^Tb]Tb-DOTA-LM3 and [^177^Lu]Lu-DOTATOC were 36.6 Gy/GBq (range, 15–196 Gy/GBq) and 7.0 Gy/GBq (range, 2.4–14.2 Gy/GBq), respectively (*P* = 0.008). The median kidney and bone marrow absorbed doses were 2.4 Gy/GBq (range, 1.8–3.1 Gy/GBq) and 0.31 Gy/GBq (range, 0.24–0.48 Gy/GBq) for [^161^Tb]Tb-DOTA-LM3 and 0.6 Gy/GBq (range, 0.4–0.8 Gy/GBq) and 0.04 Gy/GBq (range, 0.03–0.06 Gy/GBq) for [^177^Lu]Lu-DOTATOC, respectively (both *P* = 0.008). According to Common Terminology Criteria for Adverse Events version 5.0, grade 1–3 treatment-emergent adverse events occurred in 6 of 8 patients after administration of 1 GBq of [^161^Tb]Tb-DOTA-LM3. **Conclusion:** [^161^Tb]Tb-DOTA-LM3 showed a 7.6-fold-higher median tumor absorbed dose than that of [^177^Lu]Lu-DOTATOC. The tumor–to–bone marrow absorbed dose ratio was in the same range for [^161^Tb]Tb-DOTA-LM3 as for [^177^Lu]Lu-DOTATOC. The administration of 1 GBq of [^161^Tb]Tb-DOTA-LM3 was safe for all patients, without relevant adverse events.

## Visual Abstract

**Figure:**
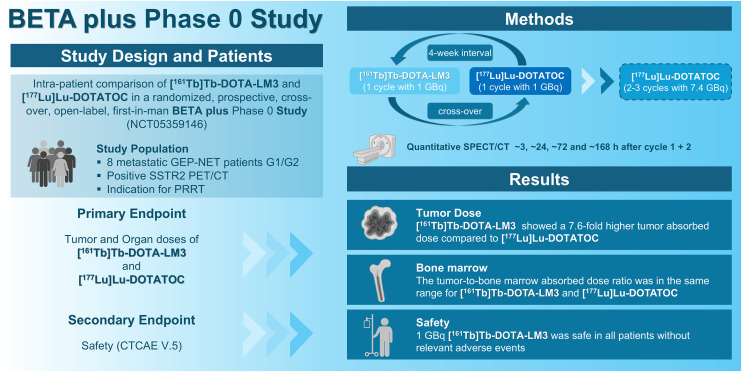

Terbium-161 is an intriguing addition to the list of commercially available radionuclides for radiopharmaceutical therapy. It shows a half-life (6.95 d) and an energy of β^−^-particles (average, 154 keV) (1) similar to those of ^177^Lu (half-life, 6.64 d; average energy of β^−^-particles, 134 keV). Moreover, ^161^Tb emits x-rays (45.2, 46.0, and 52.2 keV at 6.3%, 11%, and 3.6%, respectively) and γ-photons (e.g., 48.9 keV at 17% and 74.6 keV at 10%) that can be used for SPECT imaging and dosimetry calculations (2,3). The advantage of ^161^Tb lies in its additional emission of large numbers of short-range conversion and Auger electrons, which result in up to 4-fold-higher energy deposition, within a radius of up to about 40 µm from the decay site, than resulted from ^177^Lu. Beyond this range, the energy deposition is about the same as ^177^Lu (4). This enhanced short-range energy deposition is believed to provide a therapeutic advantage for ^161^Tb over ^177^Lu, particularly with regard to possible cell membrane damage and elimination of single cell clusters and micrometastases (5–9).

Peptide receptor radionuclide therapy (PRRT) with [^177^Lu]Lu-DOTATATE (Lutathera; Novartis) and [^177^Lu]Lu-DOTATOC is an established treatment for neuroendocrine tumors that uses the overexpression of somatostatin receptor subtype 2 (SSTR2) as a molecular target (10–12). There is clinical evidence that PRRT using α-particle emitters such as ^225^Ac is more effective than PRRT with [^177^Lu]Lu-DOTATATE (13). This is due to the high linear energy transfer of α-particles, which have the advantage of more effectively inducing DNA double-strand breaks and hence causing severe chromosomal damage, in contrast to β^−^-emitters such as ^177^Lu, which mainly cause DNA single-strand breaks. However, the longer range of β^−^-particles results in a stronger cross-fire effect than that of the short-range α-particles (14). The cross-fire effect is important for the efficacy of PRRT because of the destruction of multiple cells around a radiopharmaceutical-accumulating cell, which compensates for heterogeneous SSTR2 expression on tumor cells. ^161^Tb combines the cross-fire effect of β^−^-particles with the capability of possible cell membrane damage and elimination of single cell clusters and micrometastases with short-range conversion and Auger electrons.

Several studies have highlighted the potential benefits of radiolabeled SSTR2 antagonists such as [^177^Lu]Lu-DOTA-JR11 (synonyms [^177^Lu]Lu-OPS201 and [^177^Lu]Lu-satoreotide tetraxetan) and [^177^Lu]Lu-DOTA-LM3 over SSTR2 agonists for PRRT. They exhibit a higher number of binding sites to SSTR2 and prolonged retention on the cell membrane, resulting in higher tumor uptake and, consequently, increased radiation dose delivery to tumors compared with standard PRRT (15–21).

Studies suggest that the cell membrane is an interesting radiosensitive structure to the dense ionization caused by conversion and Auger electrons (5,22). SSTR2 antagonists do not readily internalize, with more than 90% of the radiopharmaceutical remaining localized on the cell membrane, which makes the combination of ^161^Tb with these antagonists a highly promising strategy for PRRT. The superior therapeutic effect of [^161^Tb]Tb-DOTA-LM3 was demonstrated in preclinical studies that compared the ^161^Tb- and ^177^Lu-labeled SSTR2 agonist and antagonist in AR42J tumor-bearing mice, as well as by clonogenic cell survival assays (5,23). Therefore, we hypothesize that the tumor radiation dose will be several times higher with [^161^Tb]Tb-DOTA-LM3 than with [^177^Lu]Lu-DOTATOC in patients with SSTR2-positive tumors. First-in-human administration of [^161^Tb]Tb-DOTA-LM3 showed promising results in the first patient of this study (24).

In this work, we report the results from the β^−^-particle plus conversion and Auger-electron therapy phase 0 study, which compared the tumor and relevant organ doses of [^161^Tb]Tb-DOTA-LM3 with [^177^Lu]Lu-DOTATOC in the same patients with grade 1 or 2 gastroenteropancreatic neuroendocrine tumors (NETs).

## MATERIALS AND METHODS

### Study Design and Patients

Eight patients with gastroenteropancreatic NETs were consecutively included in this prospective, randomized, crossover, single-center, open-label phase 0 dosimetry comparison study (NCT05359146, registered with ClinicalTrials.gov on March 28, 2023). The Ethics Committee of Northwest and Central Switzerland approved this study, and all patients signed an informed-consent form. The main inclusion criteria were histologically confirmed, well-differentiated, functioning, or nonfunctioning metastatic gastroenteropancreatic NETs (grades 1 and 2) with indication for PRRT and absence of a curative surgical option (assessment by the local multidisciplinary NET board); Eastern Cooperative Oncology Group status of no more than 2; and SSTR2 expression on [^68^Ga]Ga-DOTATOC or [^68^Ga]Ga-DOTATATE PET/CT imaging. The main exclusion criterion was the administration of chemotherapy in the last 4 wk before inclusion or of a therapeutic radiopharmaceutical during 8 effective half-lives of its decay. Further inclusion and exclusion criteria are listed in the supplemental materials (supplemental materials are available at http://jnm.snmjournals.org) (25–30).

### Preparation of Radiopharmaceuticals

The ^161^Tb used in this study was produced as no-carrier-added [^161^Tb]TbCl_3_ in 0.05 M HCl by the Radionuclide Development group at the Paul Scherrer Institute, as previously reported (31). [^161^Tb]Tb-DOTA-LM3 was manufactured by radiolabeling 100 µg of DOTA-LM3 with [^161^Tb]TbCl_3_ using an automated synthesis module. The ^161^Tb product was combined with DOTA-LM3 in a reaction buffer containing sodium ascorbate. The mixture was incubated at 60 °C for 20 min, purified over a C18 cartridge, and eluted with an aqueous ethanol solution. The final product was formulated in a solution containing physiologic saline, ascorbic acid, and calcium trisodium pentetate. The radiochemical purity was determined by radio–high-performance liquid chromatography and was at least 95%. Radionuclidic purity of at least 99.9% was ensured by γ-spectrometry. The release specification included limits for peptide content and corresponding metal complexes (≤100 µg/patient dose), ethanol content (≤7%), and bacterial endotoxins (<175 endotoxin units per patient dose). The integrity of the sterile filter was confirmed by a matrix-based bubble point test (≥2.86 bar).

[^177^Lu]Lu-DOTATOC was produced in a kit-labeling procedure, by adding 240 µg of DOTATOC dissolved in sodium ascorbate buffer (pH 5) to a vial containing 1 GBq of no-carrier-added [^177^Lu]LuCl_3_ (EndolucinBeta; ITM GmbH), and subsequently heated at 95 °C for 30 min. The final product was formulated in a physiologic saline solution containing calcium-diethylenetriamine pentaacetate as the radioisotope scavenger. Radiochemical purity was assessed by radio–high-performance liquid chromatography and was at least 95%. The incorporation yield was measured by radio–thin-layer chromatography with levels of unbound ^177^Lu of no more than 0.5%.

### Study Protocol and SPECT/CT Imaging

Patients received 1 GBq of [^161^Tb]Tb-DOTA-LM3 (peptide amount, ≤100 µg) as an intravenous infusion over 60 min and 1 GBq of [^177^Lu]Lu-DOTATOC (peptide amount, ∼240 µg) as an intravenous injection over 1 min. A longer infusion time was chosen for [^161^Tb]Tb-DOTA-LM3 to improve tolerability, because SSTR2 antagonists can cause transient infusion-related symptoms when administered rapidly. The 2 test injections were administered in a randomized crossover design with an approximately 4-wk interval to minimize carryover effects and bias. Both test injections were followed by 2–3 cycles of 5.6–7.4 GBq of [^177^Lu]Lu-DOTATOC (peptide amount, ∼240 µg). For kidney protection, patients received 20 g/L lysine and 20 g/L arginine in 1 L of 0.9% saline solution over 4 h, starting 1 h before administration of the radiopharmaceutical. Quantitative SPECT/CT imaging was performed for both radiopharmaceuticals using a Symbia Intevo 16 system (Siemens Healthineers). A step-and-shoot acquisition protocol was used, consisting of 60 projections at 30 s per projection, across 2 bed positions covering chest, abdomen, and pelvis. A low-energy, high-resolution collimator was used for [^161^Tb]Tb-DOTA-LM3, and a medium-energy, low-penetration collimator was used for [^177^Lu]Lu-DOTATOC (3). The energy acquisition protocol included 2 photopeak windows for [^161^Tb]Tb-DOTA-LM3 (48 keV ± 20% and 75 keV ± 10%) and 1 photopeak window for [^177^Lu]Lu-DOTATOC (208 keV ± 10%). For quantitative analysis of [^161^Tb]Tb-DOTA-LM3, only the 75 keV ± 10% window was used. The low-dose, non–contrast-enhanced CT scan was acquired using the standard parameters of 110 kV and 60 mAs.

### Dosimetry

Tumor and organ absorbed doses for [^161^Tb]Tb-DOTA-LM3 and [^177^Lu]Lu-DOTATOC were calculated from serial SPECT/CT imaging at 3, 24, 72, and 168 h after injection. The crossover design, with a 4-wk washout interval, enabled intraindividual comparison and assessment of sequencing effects, thereby minimizing bias. Reconstructions were performed using a Monte Carlo–based ordered-subset expectation maximization reconstruction algorithm with 6 iterations and 10 subsets (32). For tumor dosimetry, all delineable target lesions (≤3 target lesions) per patient were chosen on the basis of baseline somatostatin receptor (SSTR) PET/CT, with inclusion criteria of a minimum lesion volume of 1 mL. Tumor volume segmentation was performed on contrast-enhanced CT or MRI. Activity quantification was performed using volumes of interest encompassing the lesion with an added margin to account for partial-volume effects, applying a correction for surrounding background activity contributions. Masking was not necessary or possible because of the intrapatient comparison of the same target lesions with both compounds. For details, see the dosimetry in the supplemental materials.

### Toxicity Assessment

During the 60-min infusion of [^161^Tb]Tb-DOTA-LM3, vital signs such as blood pressure, heart rate, and oxygen saturation were monitored every 15 min. In addition, a full blood count and comprehensive metabolic panel were conducted before the administration of the radiopharmaceutical and at least 2, 4, and 6 wk after treatment to assess potential adverse events. Adverse events were graded using Common Terminology Criteria for Adverse Events version 5.0 (National Cancer Institute).

### Statistical Analysis

A sample size calculation was not performed in this phase 0 study. Descriptive statistics were used to present all data. To compare paired and unpaired dosimetry data between [^177^Lu]Lu-DOTATOC and [^161^Tb]Tb-DOTA-LM3, the Wilcoxon signed-rank test and the Mann–Whitney *U* test were applied as appropriate. All statistical tests were 2-tailed, and a *P* value of less than 0.05 was considered statistically significant. Unless specified otherwise, data are presented as the median with the corresponding range.

## RESULTS

### Patient Characteristics

From March 2023 until January 2024, 8 patients were included as part of the study. The demographic and clinical characteristics are presented in Table 1. All patients received 1 infusion of [^161^Tb]Tb-DOTA-LM3 at activity of 0.9–1.1 GBq (peptide amount, ≤100 µg) and 1 injection of [^177^Lu]Lu-DOTATOC at activity of 1.0–1.1 GBq (peptide amount, ∼240 µg) at an interval of 4 ± 1 wk in a crossover setting to balance possible carryover effects. Because this activity was below the commonly applied therapeutic amount, 2 patients received 2 additional treatment cycles and 6 patients received 3 additional treatment cycles of [^177^Lu]Lu-DOTATOC (7.4 GBq, ∼240 µg) based on clinical needs.

**TABLE 1.** Summary of Patient Characteristics

Characteristic	Value
*n*	8
Age (y)	66 (51–78)
Sex	
Male	4 (50)
Female	4 (50)
Time since initial diagnosis (mo)	27 (2–95)
Mean ± SD	38 ± 13
Time from last PRRT to progress (mo)	23 (14–31)
Mean ± SD	23 ± 12
ECOG	
0	7 (12.5)
1	1 (87.5)
Primary tumor type	
Gastrointestinal	3 (37.5)
Pancreatic	5 (62.5)
Tumor grade	
1	1 (12.5)
2	7 (87.5)
3	0 (0)
NET functionality	
Functioning	4 (50)
Nonfunctioning	4 (50)
Unknown	0 (0)
Ki-67 proliferation index (%)	6 (3–18)
Not evaluable (*n*)	2
Prior treatments	
Somatostatin analogs	6 (75)
Surgery	4 (50)
Chemotherapy	0 (0)
Radiotherapy	1 (12.5)
PRRT	2 (25)

ECOG = Eastern Cooperative Oncology Group.

Qualitative data are number followed by percentage in parentheses; continuous data are median followed by range in parentheses.

### Dosimetry Results and Response

Table 2 presents the results of tumor and bone marrow absorbed dose estimations, as well as tumor–to–bone marrow and tumor-to-kidney absorbed dose ratios for all 8 patients. The effective tumor half-life was significantly longer with [^161^Tb]Tb-DOTA-LM3 (half-life, 103 h; range, 56–152 h) than with [^177^Lu]Lu-DOTATOC (half-life, 83 h; range, 30–122 h), (*P* = 0.012). The median tumor absorbed dose for [^161^Tb]Tb-DOTA-LM3 was 7.6-fold higher (range, 2.7–13.8; *P* = 0.008) than for [^177^Lu]Lu-DOTATOC. The tumor-to-kidney absorbed dose ratio was 1.7-fold higher for [^161^Tb]Tb-DOTA-LM3 than for [^177^Lu]Lu-DOTATOC (range, 0.5–2.9; *P* = 0.039). In addition, the absorbed dose to the bone marrow was higher after injection of [^161^Tb]Tb-DOTA-LM3 in our patient collective (*P* = 0.008). Comparison of tumor dose ratios ([^161^Tb]Tb-DOTA-LM3/[^177^Lu]Lu-DOTATOC) between patients who received [^161^Tb]Tb-DOTA-LM3 first and those who received [^177^Lu]Lu-DOTATOC first revealed no statistically significant difference (median, 7.98 vs. 6.51; *P* = 0.686), suggesting that sequencing did not substantially influence the relative tumor dose increase observed with [^161^Tb]Tb-DOTA-LM3. Supplemental Table 1 shows a comparison of absorbed organ doses of [^177^Lu]Lu-DOTATOC and [^161^Tb]Tb-DOTA-LM3.

**TABLE 2.** Summary of Tumor, Bone Marrow, and Kidney Radiation Dose Estimations in Same Patients

	[^177^Lu]Lu-DOTATOC					[^161^Tb]Tb-DOTA-LM3				
Patient	Tumor dose* (Gy/GBq)	Red marrow dose (Gy/GBq)	Kidney dose (Gy/GBq)	T/RM ratio	T/K ratio	Tumor dose* (Gy/GBq)	Red marrow dose (Gy/GBq)	Kidney dose (Gy/GBq)	T/RM ratio	T/K ratio
1	12.6	0.03	0.6	424	23	86.0	0.31	2.5	274	35
2	10.2	0.03	0.4	321	25	27.8	0.24	2.1	115	13
3	3.4	0.06	0.6	54	6	28.0	0.40	2.8	70	10
4	5.0	0.04	0.7	124	8	45.2	0.42	3.1	107	14
5	2.4	0.03	0.5	90	5	23.4	0.29	1.8	82	13
6	9.0	0.05	0.7	166	13	63.8	0.48	2.9	132	22
7	14.2	0.03	0.5	451	29	196.3	0.26	2.3	753	84
8	3.7	0.05	0.8	69	5	15.0	0.31	2.3	48	7
Median	7.0	0.04	0.6	145	10	36.6	0.31	2.4	111	14
Range	2.4–14.2	0.03–0.06	0.4–0.8	54–451	5–29	15.0–196	0.24–0.48	1.8–3.1	48–753	7–84

* Mean tumor dose of 1–3 tumors per patient. Lesion volumes ranged from 1.04 to 22.1 mL.

T/RM = tumor–to–red marrow ratio; T/K = tumor-to-kidney ratio.

**FIGURE 1.**
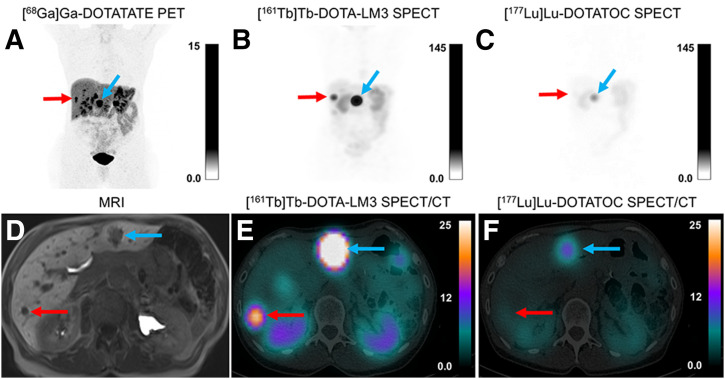
Patient 7 with well-differentiated metastatic NET of pancreas (grade 2, Ki-67 index 5%). (A–F) Baseline maximum-intensity projection of coronal PET/CT 1 h after administration of [^68^Ga]Ga-DOTATATE (A), maximum-intensity projection of coronal SPECT 72 h after administration of 1 GBq of [^161^Tb]Tb-DOTA-LM3 (B) and 72 h after administration of 1 GBq of [^177^Lu]Lu-DOTATOC (C), transaxial T1-weighted baseline MRI (D), and transaxial SPECT/CT 72 h after administration of 1 GBq of [^161^Tb]Tb-DOTA-LM3 (E) and 72 h after administration of 1 GBq of [^177^Lu]Lu-DOTATOC (F). [^161^Tb]Tb-DOTA-LM3 SPECT/CT clearly delineates both liver metastases, despite smaller one measuring only 7 mm (red arrow). In contrast, [^177^Lu]Lu-DOTATOC SPECT/CT visualizes only larger metastasis (blue arrow). Scale bars indicate SUV in A and kilobecquerels in B, C, E, and F.

**FIGURE 2.**
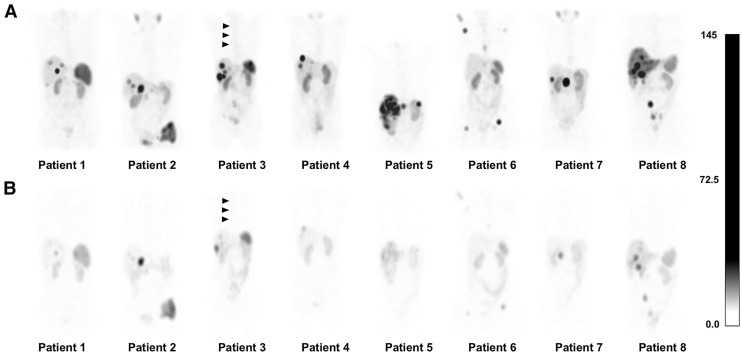
Maximum-intensity projection of coronal quantitative SPECT images acquired 72 h after administration of [^161^Tb]Tb-DOTA-LM3 (A) and [^177^Lu]Lu-DOTATOC (B) in interval of 4 ± 1 wk in crossover setting in same patients. Equal grayscale windowing was applied, with voxel values expressed in kilobecquerels. Although tumor uptake with [^161^Tb]Tb-DOTA-LM3 was higher than with [^177^Lu]Lu-DOTATOC in all patients, bone marrow uptake was also more pronounced with [^161^Tb]Tb-DOTA-LM3 than with [^177^Lu]Lu-DOTATOC (indicated by arrowheads for patient 3).

Figure 1 shows the results for patient 7. Figure 2 illustrates the maximum-intensity projection of SPECT images for [^161^Tb]Tb-DOTA-LM3 and [^177^Lu]Lu-DOTATOC for all 8 patients at 72 h. The disease control rate at 12 mo was 75% after therapy with 15.8–23.2 GBq of [^177^Lu]Lu-DOTATOC plus 1 GBq of [^161^Tb]Tb-DOTA-LM3: 3 patients achieved partial remission, and 3 patients had stable disease.

### Safety

Hematologic treatment-emergent adverse events occurred in 6 of the 8 patients who received 1 GBq of [^161^Tb]Tb-DOTA-LM3, which included anemia, neutropenia, and thrombocytopenia at grade 1; leukopenia at grades 1 and 2; and lymphopenia at grades 2 and 3 (Table 3). Nonhematologic treatment-emergent adverse events were all grade 1 and occurred in 4 of the 8 patients (fatigue, abdominal pain, hypotension, nausea, hair loss, dizziness, and headache). No grade 4 or 5 adverse events were observed. All treatment-emergent adverse events resolved within a few weeks after detection, except for 1 patient who, after presenting with grade 2 lymphopenia at baseline, developed grade 3 lymphopenia that had not recovered at the time of the last follow-up. Up to 12 mo after administration of [^161^Tb]Tb-DOTA-LM3, no decrease in kidney function and no evidence of myelodysplastic syndrome or other neoplasms were observed.

**TABLE 3.** Treatment-Emergent Adverse Events After [^161^Tb]Tb-DOTA-LM3 Administration

Adverse event	Grades 1–2	Grade 3
Any adverse event	16	1
Blood disorder		
Anemia	4 (50)	0
Neutropenia	1 (12.5)	0
Leukopenia	2 (25)	0
Lymphopenia	2 (25)	1 (12.5)
Thrombocytopenia	1 (12.5)	0
General disorder: fatigue	1 (12.5)	0
Gastrointestinal disorder: abdominal pain	1 (12.5)	0
Vascular disorder: hypotension	1 (12.5)	0
Skin disorder: hair loss	1 (12.5)	0
Nervous system disorder		
Headache	1 (12.5)	0
Dizziness	1 (12.5)	0

Data are number followed by range in parentheses.

Shown are all treatment-emergent adverse events reported in all 8 study patients. Data were collected from date of first test injection until date of first therapy with 7.4 GBq of [^177^Lu]Lu-DOTATOC. No grade 4 or 5 adverse events were observed in study population.

## DISCUSSION

This phase 0 study represents the first investigation, to our knowledge, of a ^161^Tb-labeled SSTR antagonist in humans. It provides a direct intrapatient comparison of tumor and organ absorbed doses with the established radiolabeled SSTR agonist [^177^Lu]Lu-DOTATOC (12).

The main results of this study can be summarized as follows. First, the median tumor absorbed dose was 7.6-fold higher (range, 2.7–13.8 Gy/GBq) with [^161^Tb]Tb-DOTA-LM3 than with [^177^Lu]Lu-DOTATOC. The marked and significant increase in absorbed tumor dose can be explained by the presence of additional conversion and Auger electrons (∼40% additional energy), the slightly higher mean β^−^-energy of ^161^Tb than of ^177^Lu (154 vs. 134 keV) (33), eventually the lower peptide amount of [^161^Tb]Tb-DOTA-LM3 than of [^177^Lu]Lu-DOTATOC (21,34), and most importantly, the longer retention and higher accumulation of the SSTR antagonist [^161^Tb]Tb-DOTA-LM3 than the agonist on tumor cells. These effects seem to be particularly pronounced in microscopic tumors (7,8). Second, the administration of 1 GBq of [^161^Tb]Tb-DOTA-LM3 resulted in a high median absorbed tumor dose of 37 Gy (range, 15–196 Gy). Third, additional renal toxicity is expected to be negligible with [^161^Tb]Tb-DOTA-LM3 because of a 1.7-fold-higher (median) tumor-to-kidney absorbed dose ratio than with [^177^Lu]Lu-DOTATOC. Fourth, tumor–to–bone marrow absorbed dose ratios of both radiopharmaceuticals were in the same range, although the estimated absorbed bone marrow dose was 6.2- to 10.5-fold higher with [^161^Tb]Tb-DOTA-LM3 than with [^177^Lu]Lu-DOTATOC. Lastly, the low hematologic toxicity after administration of 1 GBq of [^161^Tb]Tb-DOTA-LM3 justifies the evaluation of more cycles with increased injected activity in future phase 1 or 2 clinical studies.

These observations give rise to the expectation that [^161^Tb]Tb-DOTA-LM3 therapy results in a better tumor control rate in patients with gastroenteropancreatic NETs, because it synergistically combines the advantages of β^−^-emission (strong cross-fire effect), short-range conversion and Auger electrons, and the enhanced targeting properties of an SSTR antagonist (5,23). However, bone marrow toxicity might become a limitation for the clinical use of radiolabeled SSTR antagonists. During dose-finding clinical studies, more frequent grade 3 or greater hematologic toxicity was shown in patients treated with radiolabeled SSTR antagonists such as [^177^Lu]Lu-DOTA-LM3 and [^177^Lu]Lu-DOTA-JR11 (19,20,35) than in patients treated with radiolabeled SSTR agonists such as [^177^Lu]Lu-DOTATATE and [^177^Lu]Lu-DOTATOC (10–12). Previous studies have demonstrated that human hematopoietic cells, particularly CD34 + progenitor cells, express SSTR2 (36,37). This may explain the enhanced hematotoxicity observed with [^177^Lu]Lu-DOTA-JR11 and [^177^Lu]Lu-DOTA-LM3, because both radiopharmaceuticals exhibit a greater ability than [^177^Lu]Lu-DOTATOC to bind to SSTR2-expressing hematopoietic stem and progenitor cells (37). Given the specific accumulation of radiolabeled SSTR antagonists in the bone marrow, blood-based bone marrow dosimetry may underestimate toxicity risks (34). Therefore, imaging-based dosimetry should be favored.

There is preclinical evidence that an increased peptide quantity of the antagonist may reduce the bone marrow radiation dose by saturating SSTR2, thereby improving the therapeutic index (18). In our study, peptide amounts of no more than 100 µg of DOTA-LM3 and approximately 240 µg of DOTATOC were used, resulting in specific activity of approximately 17 GBq/µmol for approximately 1 GBq of [^161^Tb]Tb-DOTA-LM3 and approximately 7 GBq/µmol for approximately 1 GBq of [^177^Lu]Lu-DOTATOC. The lower molar activity of [^177^Lu]Lu-DOTATOC could have contributed to a higher tumor–to–bone marrow dose ratio.

Several limitations of this study should be acknowledged. First, the small sample size may limit the generalizability of the findings. Second, high tumor radiation doses of more than 60 Gy in 3 patients after administration of only 1 GBq of [^161^Tb]Tb-DOTA-LM3 may have a carryover effect (therapeutic effect with a decrease of SSTR2-expressing tumor cells) onto [^177^Lu]Lu-DOTATOC, resulting in a decrease of the tumor radiation dose; however, the crossover design allowed assessment of this potential carryover effect and did not reveal any significant difference. Third, treatment of all patients with standard [^177^Lu]Lu-DOTATOC therapy just after crossover administration of the 2 radiopharmaceuticals prevented a comprehensive evaluation of potential late toxicities and therapeutic outcomes. Finally, the influence of the peptide amount on the therapeutic index and safety was not systematically assessed, representing an area for future research.

## CONCLUSION

[^161^Tb]Tb-DOTA-LM3 shows a 7.6-fold-higher tumor absorbed dose than that found when using [^177^Lu]Lu-DOTATOC. The tumor–to–bone marrow absorbed dose ratio was in the same range for both radiopharmaceuticals. The promising dosimetry and safety results from this phase 0 study will lay the foundation for further clinical development of [^161^Tb]Tb-DOTA-LM3. Dose-escalation trials and peptide dose optimizing studies are warranted to establish the maximum tolerated activity per cycle and assess long-term efficacy. The integration of ^161^Tb into PRRT regimens has the potential to redefine the therapeutic landscape for NETs, particularly for patients with suboptimal responses to current treatments or possible treatment of micrometastases.

## DISCLOSURE

The study was supported by the Swiss National Science Foundation (number 32003B_205070), which had no role in the conduct of the study, with funding directed to Damian Wild, Roger Schibli, and Emanuel Christ. Peter Bernhardt acknowledges funding from the Swedish Cancer Society, the Jubilee Clinic Cancer Research Foundation, and the ALF agreement. Cristina Müller, Roger Schibli, Nicholas van der Meulen, Melpomeni Fani, and Damian Wild are listed as inventors on U.S. patent application US2023/0165981, which contains [^161^Tb]Tb-DOTA-LM3. Peter Bernhardt is a cofounder of Theravision AB. No other potential conflict of interest relevant to this article was reported.

## Notes

KEY POINTS **QUESTION:** Can [^161^Tb]Tb-DOTA-LM3 provide higher tumor absorbed doses and maintain a more favorable bone marrow absorbed dose than [^177^Lu]Lu-DOTATOC in patients with metastatic gastroenteropancreatic NETs? **PERTINENT FINDINGS:** In this single-center, phase 0 study, [^161^Tb]Tb-DOTA-LM3 demonstrated a 7.6-fold-higher median tumor absorbed dose coefficient than that of [^177^Lu]Lu-DOTATOC. Tumor–to–bone marrow absorbed dose ratios were in the same range for both radiopharmaceuticals, and no relevant hematologic toxicity was observed after the administration of 1 GBq of [^161^Tb]Tb-DOTA-LM3. **IMPLICATIONS FOR PATIENT CARE:** [^161^Tb]Tb-DOTA-LM3 may enhance tumor absorbed doses while maintaining a tolerable safety profile, supporting further clinical development in patients with metastatic gastroenteropancreatic NETs.

## References

[1] Duran MT Juget F Nedjadi Y. Determination of ^161^Tb half-life by three measurement methods. Appl Radiat Isot. 2020;159:109085. 32250758 10.1016/j.apradiso.2020.109085

[2] Lehenberger S Barkhausen C Cohrs S. The low-energy β^−^ and electron emitter ^161^Tb as an alternative to ^177^Lu for targeted radionuclide therapy. Nucl Med Biol. 2011;38:917–924. 21843788 10.1016/j.nucmedbio.2011.02.007

[3] Marin I Ryden T Van Essen M. Establishment of a clinical SPECT/CT protocol for imaging of ^161^Tb. EJNMMI Phys. 2020;7:45. 32613587 10.1186/s40658-020-00314-x PMC7329978

[4] Westerbergh F. Image-based dosimetry in targeted radionuclide therapy with terbium-161: from technical foundations to clinical application [doctoral thesis]. Gothenburg University Library website. https://gupea.ub.gu.se/handle/2077/85342. Updated May 15, 2025. Accessed November 24, 2025.

[5] Spoormans K Struelens L Vermeulen K. The emission of internal conversion electrons rather than Auger electrons increased the nucleus-absorbed dose for ^161^Tb compared with ^177^Lu with a higher dose response for [^161^Tb]Tb-DOTA-LM3 than for [^161^Tb]Tb-DOTATATE. J Nucl Med. 2024;65:1619–1625. 39209546 10.2967/jnumed.124.267873

[6] Ku A Facca VJ Cai Z. Auger electrons for cancer therapy: a review. EJNMMI Radiopharm Chem. 2019;4:27. 31659527 10.1186/s41181-019-0075-2 PMC6800417

[7] Bernhardt P Benjegard SA Kolby L. Dosimetric comparison of radionuclides for therapy of somatostatin receptor-expressing tumors. Int J Radiat Oncol Biol Phys. 2001;51:514–524. 11567828 10.1016/s0360-3016(01)01663-7

[8] De Nardo L Santi S Dalla Pieta A. Comparison of the dosimetry and cell survival effect of ^177^Lu and ^161^Tb somatostatin analog radiopharmaceuticals in cancer cell clusters and micrometastases. EJNMMI Phys. 2024;11:94. 39535653 10.1186/s40658-024-00696-2 PMC11561253

[9] Hindie E Larouze A Alcocer-Avila M. Palladium-103 (^103^Pd/^103m^Rh), a promising Auger-electron emitter for targeted radionuclide therapy of disseminated tumor cells: absorbed doses in single cells and clusters, with comparison to ^177^Lu and ^161^Tb. Theranostics. 2024;14:4318–4330. 39113794 10.7150/thno.95436 PMC11303077

[10] Strosberg J El-Haddad G Wolin E.; NETTER-1 Trial Investigators. Phase 3 trial of ^177^Lu-DOTATATE for midgut neuroendocrine tumors. N Engl J Med. 2017;376:125–135. 28076709 10.1056/NEJMoa1607427 PMC5895095

[11] Singh S Halperin D Myrehaug S.; NETTER-2 Trial Investigators. [^177^Lu]Lu-DOTA-TATE plus long-acting octreotide versus high-dose long-acting octreotide for the treatment of newly diagnosed, advanced grade 2–3, well-differentiated, gastroenteropancreatic neuroendocrine tumours (NETTER-2): an open-label, randomised, phase 3 study. Lancet. 2024;403:2807–2817. 38851203 10.1016/S0140-6736(24)00701-3

[12] COMPETE trial data shows ^177^Lu-edotreotide improves progression-free survival in SSTR-GEP-NETs. Applied Radiology website. https://appliedradiology.com/articles/compete-trial-data-shows-177lu-edotreotide-improves-progression-free-survival-in-sstr-gep-nets. Published March 13, 2025. Accessed November 24, 2025.

[13] Ballal S Yadav MP Tripathi M. Survival outcomes in metastatic gastroenteropancreatic neuroendocrine tumor patients receiving concomitant ^225^Ac-DOTATATE targeted α-therapy and capecitabine: a real-world scenario management based long-term outcome study. J Nucl Med. 2023;64:211–218. 35863893 10.2967/jnumed.122.264043

[14] Haberkorn U Giesel F Morgenstern A. The future of radioligand therapy: α, β, or both? J Nucl Med. 2017;58:1017–1018. 28408527 10.2967/jnumed.117.190124

[15] Mansi R Plas P Vauquelin G. Distinct in vitro binding profile of the somatostatin receptor subtype 2 antagonist [^177^Lu]Lu-OPS201 compared to the agonist [^177^Lu]Lu-DOTA-TATE. Pharmaceuticals (Basel). 2021;14:1265. 34959665 10.3390/ph14121265 PMC8706879

[16] Wild D Fani M Fischer R. Comparison of somatostatin receptor agonist and antagonist for peptide receptor radionuclide therapy: a pilot study. J Nucl Med. 2014;55:1248–1252. 24963127 10.2967/jnumed.114.138834

[17] Dalm SU Nonnekens J Doeswijk GN. Comparison of the therapeutic response to treatment with a ^177^Lu-labeled somatostatin receptor agonist and antagonist in preclinical models. J Nucl Med. 2016;57:260–265. 26514177 10.2967/jnumed.115.167007

[18] Nicolas GP Mansi R McDougall L. Biodistribution, pharmacokinetics, and dosimetry of ^177^Lu-, ^90^Y-, and ^111^In-labeled somatostatin receptor antagonist OPS201 in comparison to the agonist ^177^Lu-DOTATATE: the mass effect. J Nucl Med. 2017;58:1435–1441. 28450554 10.2967/jnumed.117.191684

[19] Baum RP Zhang J Schuchardt C. First-in-humans study of the SSTR antagonist ^177^Lu-DOTA-LM3 for peptide receptor radionuclide therapy in patients with metastatic neuroendocrine neoplasms: dosimetry, safety, and efficacy. J Nucl Med. 2021;62:1571–1581. 33674401 10.2967/jnumed.120.258889 PMC8612334

[20] Wild D Grønbæk H Navalkissoor S. A phase I/II study of the safety and efficacy of [^177^Lu]Lu-satoreotide tetraxetan in advanced somatostatin receptor-positive neuroendocrine tumours. Eur J Nucl Med Mol Imaging. 2023;51:183–195. 37721581 10.1007/s00259-023-06383-1 PMC10684626

[21] Eigler C McDougall L Bauman A. Radiolabeled somatostatin receptor antagonist versus agonist for peptide receptor radionuclide therapy in patients with therapy-resistant meningioma: PROMENADE phase 0 study. J Nucl Med. 2024;65:573–579. 38423782 10.2967/jnumed.123.266817

[22] Pouget JP Santoro L Raymond L. Cell membrane is a more sensitive target than cytoplasm to dense ionization produced by Auger electrons. Radiat Res. 2008;170:192–200. 18666820 10.1667/RR1359.1

[23] Borgna F Haller S Rodriguez JMM. Combination of terbium-161 with somatostatin receptor antagonists: a potential paradigm shift for the treatment of neuroendocrine neoplasms. Eur J Nucl Med Mol Imaging. 2022;49:1113–1126. 34625828 10.1007/s00259-021-05564-0 PMC8921065

[24] Fricke J Westerbergh F McDougall L. First-in-human administration of terbium-161-labelled somatostatin receptor subtype 2 antagonist ([^161^Tb]Tb-DOTA-LM3) in a patient with a metastatic neuroendocrine tumour of the ileum. Eur J Nucl Med Mol Imaging. 2024;51:2517–2519. 38448550 10.1007/s00259-024-06641-w PMC11178597

[25] Morphis M van Staden JA du Raan H Ljungberg M. Modelling of energy-dependent spectral resolution for SPECT Monte Carlo simulations using SIMIND. Heliyon. 2021;7:e06097. 33659726 10.1016/j.heliyon.2021.e06097 PMC7892923

[26] Hemmingsson J Svensson J Hallqvist A Smits K Johanson V Bernhardt P. Specific uptake in the bone marrow causes high absorbed red marrow doses during [^177^Lu]Lu-DOTATATE treatment. J Nucl Med. 2023;64:1456–1462. 37290797 10.2967/jnumed.123.265484 PMC10478826

[27] Hemmingsson J Svensson J van der Meulen NP Muller C Bernhardt P. Active bone marrow S-values for the low-energy electron emitter terbium-161 compared to S-values for lutetium-177 and yttrium-90. EJNMMI Phys. 2022;9:65. 36153386 10.1186/s40658-022-00495-7 PMC9509518

[28] Hough M Johnson P Rajon D Jokisch D Lee C Bolch W. An image-based skeletal dosimetry model for the ICRP reference adult male: internal electron sources. Phys Med Biol. 2011;56:2309–2346. 21427487 10.1088/0031-9155/56/8/001 PMC3942888

[29] O’Reilly SE DeWeese LS Maynard MR. An image-based skeletal dosimetry model for the ICRP reference adult female: internal electron sources. Phys Med Biol. 2016;61:8794–8824. 27897136 10.1088/1361-6560/61/24/8794 PMC6385869

[30] Eckerman K Endo A. Nuclear decay data for dosimetric calculations. ICRP publication 107. Ann ICRP. 2008;38:7–96. 19285593 10.1016/j.icrp.2008.10.004

[31] Gracheva N Muller C Talip Z. Production and characterization of no-carrier-added ^161^Tb as an alternative to the clinically-applied ^177^Lu for radionuclide therapy. EJNMMI Radiopharm Chem. 2019;4:12. 31659528 10.1186/s41181-019-0063-6 PMC6620226

[32] Ryden T Heydorn Lagerlof J Hemmingsson J. Fast GPU-based Monte Carlo code for SPECT/CT reconstructions generates improved ^177^Lu images. EJNMMI Phys. 2018;5:1. 29302810 10.1186/s40658-017-0201-8 PMC5754277

[33] Verburg FA de Blois E Koolen S. Replacing Lu-177 with Tb-161 in DOTA-TATE and PSMA-617 therapy: potential dosimetric implications for activity selection. EJNMMI Phys. 2023;10:69. 37947917 10.1186/s40658-023-00589-w PMC10638215

[34] Schurrle SB Eberlein U Ansquer C. Dosimetry and pharmacokinetics of [^177^Lu]Lu-satoreotide tetraxetan in patients with progressive neuroendocrine tumours. Eur J Nucl Med Mol Imaging. 2024;51:2428–2441. 38528164 10.1007/s00259-024-06682-1 PMC11178655

[35] Reidy-Lagunes D Pandit-Taskar N O’Donoghue JA. Phase I trial of well-differentiated neuroendocrine tumors (NETs) with radiolabeled somatostatin antagonist ^177^Lu-satoreotide tetraxetan. Clin Cancer Res. 2019;25:6939–6947. 31439583 10.1158/1078-0432.CCR-19-1026 PMC8382090

[36] Oomen SP van Hennik PB Antonissen C. Somatostatin is a selective chemoattractant for primitive (CD34^+^) hematopoietic progenitor cells. Exp Hematol. 2002;30:116–125. 11823046 10.1016/s0301-472x(01)00772-x

[37] Nguyen N Min Y Riviere J. Limitations of the radiotheranostic concept in neuroendocrine tumors due to lineage-dependent somatostatin receptor expression on hematopoietic stem and progenitor cells. Theranostics. 2025;15:6497–6515. 40521188 10.7150/thno.113354 PMC12160025

